# Supplementary material for: Arrhenius Crossover Temperature of Glass-Forming Liquids Predicted by an Artificial Neural Network
Source: Materials (Basel). 2023 Jan 28;16(3):1127. doi: 10.3390/ma16031127 (PMC9921151; doi:10.3390/ma16031127)
Supplement: Supplementary file 1 [file materials-16-01127-s001.zip › supporting_information.pdf]

Arrhenius Crossover Temperature of Glass-Forming Liquids Predicted by an Artificial Neural Network

Bulat N. Galimzyanov [1,2], Maria A. Doronina [1] and Anatolii V. Mokshin [1,2]

[1]Institute of Physics, Kazan Federal University 420008 Kazan, Russia;
[2]Udmurt Federal Research Center of the Ural Branch of RAS, 426067 Izhevsk, Russia

Table S1:Physical properties of glass-forming liquids: glass transition temperature T_g, melting temperature T_m, fragility index m, predicted $T_A^{(pred)}$ and empirical $T_A^{(emp)}$ Arrhenius crossover temperature.

No	System	T_g, K	T_m, K	m	$T_A^{(pred)}$, K	$T_A^{(emp)}$, K
1	SiO_2	1450	2000	17.9	2007	-
2	$Li_2O{\cdot}SiO_2$	593	1573	33.9	1633	-
3	$Li_2O{\cdot}2SiO_2$	727	1306	34.7	1495	-
4	$Li_2O{\cdot}3SiO_2$	734	1306	26.3	1508	-
5	$Na_2O{\cdot}SiO_2$	687	1362	40.3	1523	-
6	$Na_2O{\cdot}2SiO_2$	728	1146	35.9	1330	-
7	$Na_2O{\cdot}3SiO_2$	743	1084	26.7	1288	-
8	$Na_2O{\cdot}4SiO_2$	765	1423	29.8	1579	-
9	$K_2O{\cdot}SiO_2$	675	1248	44.2	1409	-
10	$K_2O{\cdot}2SiO_2$	768	1313	30.3	1515	-
11	$K_2O{\cdot}3SiO_2$	760	1353	24.6	1545	-
12	$K_2O{\cdot}4SiO_2$	766	1408	24.8	1574	-
13	$15.45Na_2O{\cdot}12.81CaO{\cdot}71.74SiO_2$	820	1283	22	1544	-
14	$2MgO{\cdot}2Al_2O_3{\cdot}5SiO_2$	1088	1740	35.2	1911	-
15	$2BaO{\cdot}TiO_2{\cdot}2SiO_2$	983	1714	70.5	1911	-
16	$PbO{\cdot}SiO_2$	673	1037	51.8	1203	-
17	$PbO{\cdot}2SiO_2$	749	1190	34.9	1387	-
18	$2PbO{\cdot}SiO_2$	613	1016	78.1	1164	-
19	$CaO{\cdot}MgO{\cdot}2SiO_2$	995	1664	59	1758	1839
20	$Na_2O{\cdot}Al_2O_3{\cdot}6SiO_2$	1087	1393	24	1577	1503
21	$CaO{\cdot}Al_2O_3{\cdot}2SiO_2$	1113	1825	54	2001	2010
22	B_2O_3	530	723	36	994	1073
23	$Li_2O{\cdot}B_2O_3$	693	1116	77.7	1225	-
24	$Li_2O{\cdot}2B_2O_3$	763	1190	88.2	1285	-
25	$Li_2O{\cdot}3B_2O_3$	768	1155	76.6	1272	-

Table S1: Continue.

No	System	T_g, K	T_m, K	m	$T_A^{(pred)}$, K	$T_A^{(emp)}$, K
26	$Li_2O{\cdot}4B_2O_3$	751	1108	73.8	1234	-
27	$Na_2O{\cdot}2B_2O_3$	748	1015	82.1	1176	-
28	$Na_2O{\cdot}3B_2O_3$	746	1039	65.2	1199	-
29	$Na_2O{\cdot}4B_2O_3$	727	1087	65.2	1226	-
30	$K_2O{\cdot}2B_2O_3$	705	1063	59.5	1214	-
31	$K_2O{\cdot}3B_2O_3$	709	1140	62.2	1270	-
32	$K_2O{\cdot}4B_2O_3$	691	1130	47.7	1282	-
33	$Cs_2O{\cdot}3B_2O_3$	693	1110	57.1	1249	-
34	$BaO{\cdot}2B_2O_3$	810	1183	72.4	1319	-
35	$SrO{\cdot}2B_2O_3$	911	1270	97	1404	-
36	$PbO{\cdot}B_2O_3$	658	983	80.8	1162	-
37	$PbO{\cdot}2B_2O_3$	738	1048	59.8	1209	-
38	$PbO{\cdot}3B_2O_3$	728	1023	76.5	1181	-
39	benzophenone	208	321	125	339	328
40	dibutyl phthalate	179	238	75	282	241
41	diethyl phthalate	185	270	70	282	262
42	dimethyl phthalate	195	275	70	282	261
43	dioctyl phthalate	187	223	60	281	251
44	dipropyl eneglycol	196	234	66	283	268
45	bisphenol A diglycidyl ether	255	325	96	366	309
46	glycerol	191	293	53	281	338
47	cresolphthalein-dimethyl ether	318	387	73	412	461
48	m-tricresyl phosphate	208	299	76	284	270
49	o-terphenyl	243	329	81	311	341
50	3-styrene	237	242	72	326	314
51	phenolphthalein dimethyl ether	294	373	85	399	397
52	propylene glycol	164	214	52	292	321
53	salol	221	315	73	286	309
54	sucrose benzonate	340	373	94	431	421
55	tricresyl phosphate	209	240	76	293	280
56	tripropyl eneglycol	192	232	72	284	251
57	triphenyl phosphite	204	296	69	282	286
58	xylitol	250	367	87	326	311
59	2-methy lpyridine	133	203	72	281	250
60	o-toluidine	189	250	98	296	279
61	propylene carbonate	160	224	99	285	290
62	propylbenzene	122	174	55	281	240
63	m-Fluoroaniline	173	271	70	281	312
64	polybutadiene	176	353	75	281	314
65	biphenyl	245	342	96	341	330

Table S1: Continue.

No	System	T_g, K	T_m, K	m	$T_A^{(pred)}$, K	$T_A^{(emp)}$, K
66	sucrose benzoate	340	373	94	431	421
67	trehalose	380	473	54	494	498
68	d-fructose	286	418	70	342	433
69	$Cu_{64}Zr_{36}$	800	1230	36	1495	1368
70	$Cu_{60}Zr_{20}Ti_{20}$	647	1126	41	1220	1214
71	$Cu_{50}Zr_{42.5}Ti_{7.5}$	677	1152	48	1263	1287
72	$Cu_{50}Zr_{45}Al_5$	701	1173	71	1299	1280
73	$Cu_{50}Zr_{50}$	651	1226	60	1349	1390
74	$Cu_{46}Zr_{42}Al_7Y_5$	675	1123	49	1229	1324
75	$Zr_{74}Rh_{26}$	729	1503	40	1572	1557
76	$Zr_{50}Cu_{48}Al_2$	675	1220	46	1372	1384
77	$Zr_{57}Ni_{43}$	722	1450	33	1568	1738
78	$Zr_{58.5}Cu_{15.6}Ni_{12.8}Al_{10.3}Nb_{2.8}$	672	1125	45	1230	1242
79	$Zr_{80}Pt_{20}$	715	1364	45	1550	1549
80	$Zr_{51}Cu_{36}Ni_4Al_9$	640	1173	45	1265	1300
81	$Zr_{41.2}Ti_{13.8}Cu_{12.5}Ni_{10}Be_{22.5}$	617	1026	50	1169	1144
82	$Zr_{46.75}Ti_{8.25}Cu_{7.5}Ni_{10}Be_{27.5}$	625	1050	44	1175	1205
83	$Zr_{64}Ni_{36}$	600	1283	95	1375	1400
84	$Ti_{40}Zr_{10}Cu_{36}Pd_{14}$	669	1185	64	1295	1313
85	$Ti_{40}Zr_{10}Cu_{30}Pd_{20}$	687	1279	41	1477	1472
86	$Pd_{77.5}Cu_6Si_{16.5}$	632	1058	64	1177	1160
87	$Pd_{40}Ni_{40}P_{20}$	560	1030	50	1019	1157
88	$Pd_{82}Si_{18}$	648	1071	106	1177	1186
89	$Ni_{34}Zr_{66}$	760	1283	24	1525	1506
90	$Ni_{59.5}Nb_{40.5}$	930	1448	136	1618	1658
91	$Ni_{60}Nb_{34.8}Sn_{5.2}$	890	1363	60	1569	1545
